# Angiogenesis is Inhibited by Arsenic Trioxide Through Downregulation of the CircHIPK3/miR-149-5p/FOXO1/VEGF Functional Module in Rheumatoid Arthritis

**DOI:** 10.3389/fphar.2021.751667

**Published:** 2021-10-27

**Authors:** Juan Zhang, Yeye Ma, Yue Zhang, Sijia Niu, Maolin Chu, Zhiyi Zhang

**Affiliations:** ^1^ Department of Rheumatology, The First Affiliated Hospital, Harbin Medical University, Harbin, China; ^2^ Department of Urology, The Second Affiliated Hospital, Harbin Medical University, Harbin, China

**Keywords:** circHIPK3, FoxO1, VEGF, rheumatoid arthritis, angiogenesis, inflammation

## Abstract

Angiogenesis is a crucial event in the pathogenesis of rheumatoid arthritis (RA). Arsenic trioxide (ATO, As_2_O_3_) has been reported to inhibit synovial angiogenesis *via* the vascular endothelial growth factor (VEGF)-centered functional module. However, the exact mechanisms of ATO on VEGF modulation remain unclear. Circular RNAs (circRNAs) are emerging as important regulators in RA, and the detailed mechanisms remain largely unknown. Here, we reported a circRNA (circHIPK3), the expression of which was significantly increased in RA fibroblast-like synoviocytes (RA-FLS) after TNF-α induction. Moreover, VEGF content in the supernatants of a RA-FLS and human dermal microvascular endothelial cell (HDMEC) co-culture as well as in RA-FLS co-cultured was significantly elevated in accordance with circHIPK3 levels. This increased VEGF expression may significantly upregulate endothelial tube formation and transwell migration, as well as microvessel sprouting in the *ex vivo* aortic ring assay. CircHIPK3 was further illustrated to be a sponge for the forkhead box transcription factor O1 (FOXO1)-targeting miR-149-5p, leading to the changing expression of the downstream VEGF. These networked factors mainly form a functional module regulating angiogenesis in RA-FLS, and the expression of this functional module could be significantly downregulated by ATO with a consistently reduced vascularity *in vitro*. In the collagen-induced arthritis (CIA) mice model, an intra-articular injection of the adeno-associated virus-si-circHIPK3 or ATO was demonstrated to alleviate the synovial VEGF expression and arthritis severity respectively. Thus, we elucidate a previously unknown mechanism between circRNAs and RA, and ATO has a significant protective effect on RA-FLS and CIA synovium *via* its inhibition of the angiogenic functional module of circHIPK3/miR-149-5p/FOXO1/VEGF, suggesting great potential for the combination therapy of ATO with circHIPK3 silencing.

## Introduction

Rheumatoid arthritis (RA) is a chronic inflammatory disease with a high deformity rate (Dougados et al., 2014). Although anti-TNF biologics or small-molecule drugs have been proven to be effective on some RA patients, concerns have been raised about the unresponsiveness, possible increased infection incidence, and development of malignancies with such treatments. RA is characterized by the pannus development. In our previous studies, dysregulated autophagy (Wang et al., 2019) and dysimmunity (Li et al., 2019) were verified to be involved in the progression of the RA synovial pannus formation; in particular, angiogenesis and the interaction between fibroblast-like synoviocytes (FLS) and human dermal microvascular endothelial cells (HDMECs) *via* vascular endothelial growth factor (VEGF) angiogenic functional modules play a critical role in RA disease progression (Zhang et al., 2017). A systematic study of in-depth mechanisms of vascular endothelial growth factor (VEGF) functional modules and their influence on neovascularization in RA is lacking but a must toward targeting them for clinical application.

Circular RNAs (circRNAs), a new class of noncoding RNAs, have a covalently closed loop structure with neither a 5′ cap nor a 3’ polyadenylated tail (Liu et al., 2019). Most circRNAs, which are conserved among species, exhibit higher stability than linear mRNAs (Kristensen et al., 2019). Concerning their function, circRNAs generally act as sponges for microRNAs (miRNAs) to regulate gene expression (Chen, 2020). The excellent function of circRNAs has been discovered gradually in recent years, and due to the tissue specificity and structural stability, circRNAs have become a hotspot in the field of life science. Several studies have demonstrated that circRNAs are involved in angiogenesis during the development of diseases, such as cancers (Goodall and Wickramasinghe, 2021) or myocardial infarction (Altesha et al., 2019). However, the potential role of circRNAs in the autoimmune diseases such as RA and its relevant inflammation-induced angiogenesis remains largely uninvestigated. CircHIPK3 (hsa_circ_0000284), circRNA homeodomain-interacting protein kinase 3, is recently found to be associated with human cancers and exerts specific biological effects, including the regulation of cell proliferation and migration (Zhang et al., 2020). It has also been reported that circHIPK3 could function as a miRNA sponge to regulate vascular dysfunction and vessel growth (Shan et al., 2017). In the present study, circHIPK3 was confirmed to be significantly upregulated in RA-FLS with the presence of tumor necrosis factor (TNF-α) treatment, accompanied with significant increase of VEGF expression and thus its induced vascularity. Meanwhile, miR-149-5p was predicted to be the corresponding binding miRNA according to the database (CircInteractome). More interestingly, we first demonstrated that circHIPK3 might act *via* miR-149-5p regulation of forkhead box transcription factor O1 (FOXO1) and increase VEGF expression and thus its induced RA synovial angiogenesis *in vitro and in vivo*.

Arsenic trioxide (ATO, As_2_O_3_) has attracted global attention because of its substantial anticancer activity in patients (Platzbecker et al., 2017; Zhang et al., 2018; Zhang et al., 2019a) and murine solid tumors (Griffin et al., 2000). Our previous study showed that ATO could exhibit antirheumatic effects *via* the regulation of dysregulated autophagy (Wang et al., 2019) and dysimmunity (Li et al., 2019); in particular, ATO was verified to operate an anti-angiogenic effect through the VEGF-centered functional module and significantly improved arthritis both in cell and the CIA mice model (Zhang et al., 2017). Whether circRNAs are involved in the molecular mechanisms underlying the beneficial effect of ATO on diseases remains elusive. Thus, in this study, we especially tested whether ATO has therapeutic ability through the modulation of circRNAs as well as the relevant mechanisms. In particular, we explored whether ATO exhibit the antirheumatic effect through the circHIPK3/miR-149-5p/FOXO1/VEGF functional module to inhibit synovial angiogenesis.

## Results

### CircHIPK3, but not Linear HIPK3, Was Upregulated in Human RA-FLS Induced by TNF-α, and Knockdown of circHIPK3 Inhibited the VEGF Production of RA-FLS and its Induced Angiogenesis

**FIGURE 1:**
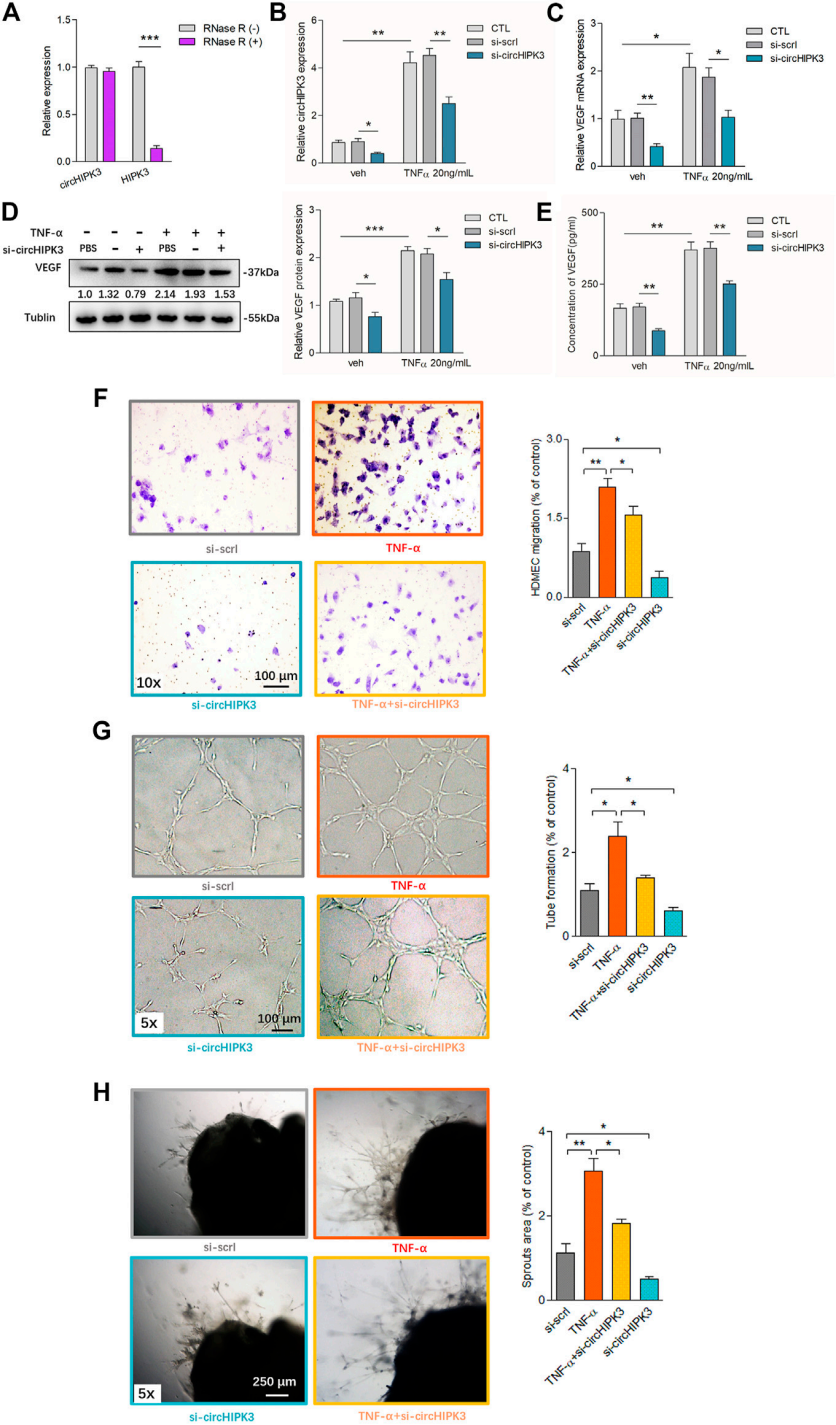
Overexpression of circHIPK3 in RA-FLS induced by TNF-α, and knockdown of circHIPK3 inhibited the VEGF production of RA-FLS. **(A)** CircHIPK3 could resist the digestion of Rnase R (n = 3; ***p < 0.001). **(B)** The expression of circHIPK3 could be significantly upregulated by TNF-α induction for 48 h, and significantly reduced by circHIPK3 knockdown whether TNF-α addition was performed or not (n = 3; *p < 0.05, **p < 0.01). **(C–E)** The effects of circHIPK3 silencing on VEGF mRNA **(C)** and protein **(D)** expression in co-cultured RA-FLS; VEGF concentration in co-culture’s supernatants **(E)** were determined by qRT-PCR, Western blot, and ELISA, respectively. VEGF expression was significantly upregulated after TNF-α induction, and significantly downregulated by circHIPK3 knockdown compared to those from the control group whether TNF-α was added or not (n = 3; *p < 0.05, **p < 0.01, ***p < 0.001). **(F–H)** The transwell assay **(F)** and tube formation test **(G)** for 6 h as well as *ex vivo* aortic ring angiogenesis assay **(H)** demonstrated significant changes in migration, the capillary-like structure formation of HDMECs and microvessel sprouting, respectively, in accordance with VEGF expression in supernatants from RA-FLS and HDMEC co-cultures (n = 3; *p < 0.05, **p < 0.01). Results are expressed as the mean ± S.E.M. Veh = vehicle control. CTL = PBS control group. si-scrl = scramble siRNA. TNF-α = 50 ng/ml.

In contrast to the linear isoforms of HIPK3 mRNA, circHIPK3 was resistant to RNase R (Figure 1A). Fluorescence *in situ* hybridization (FISH) analysis showed that circHIPK3 was located mostly in the cytoplasm of RA-FLS (Supplementary Figure S1), and it suggested that the circHIPK3 might function as an miRNA sponge. To mimic the (strengthened) microenvironment of the synovial tissue, we set up the RA-FLS and HDMEC co-culture system in the transwell apparatus besides the presence of TNF-α. To further clarify the relationship of circHIPK3 with angiogenesis under synovial inflammatory microenvironment, experiments with circHIPK3 knockdown were carefully performed. The knockdown efficiency of circHIPK3 was determined by qRT–PCR, and 60% knockdown efficiency was achieved, while the expression of linear HIPK3 mRNA was not significantly influenced (Supplementary Figure S2A). The expression level of circHIPK3 was significantly upregulated after TNF-α induction relative to that in control RA-FLS, and this increment could be obviously reduced by the knockdown of circHIPK3 (Figure 1B). Similar changes of VEGF mRNA and protein expression in co-cultured RA-FLS and the VEGF secretion in the supernatants of RA-FLS and HDMECs co-culture were observed, which were in accordance with the expression level of circHIPK3 (Figures 1C–E). Next, we asked if the changed expression of VEGF in human RA-FLS co-culture’s supernatant would have angiogenic activity, and the co-culture’s supernatants were transferred to HDMECs. The transwell assay and tube formation test demonstrated significant upregulation in the migration and capillary-like structure formation of HDMECs, respectively, under the treatment of supernatants from RA-FLS and HDMEC co-culture after TNF-α induction when compared with those from co-culture without TNF-α induction; and the migration and tube formation were significantly decreased in the circHIPK3 knockdown RA-FLS co-culture group whether TNF-α induction was performed or not (Figures 1F,G,I). Interestingly, mouse aortic rings *ex vivo* showed similar changing trends with the results of the transwell assay and tube formation test (Figures 1H,I). These data suggest that knockdown of circHIPK3 impaired the RA-FLS induced synovial angiogenesis *via* regulating the expression of VEGF.

### Interaction of CircHIPK3 With miR-149-5p, and FOXO1 Was the Further Target

**FIGURE 2:**
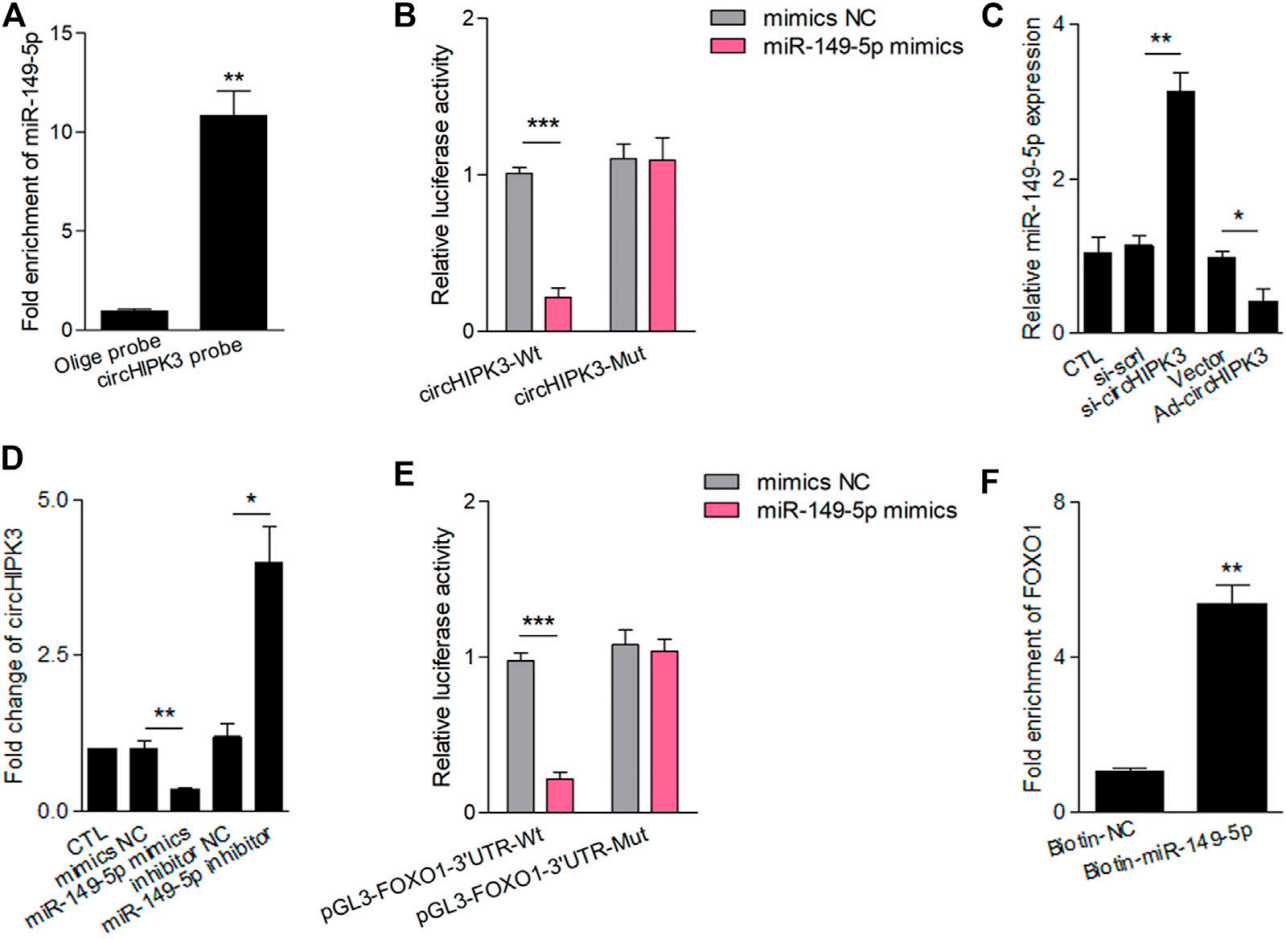
CircHIPK3 interacted with miR-149-5p, and FOXO1 was a target of miR-149-5p. **(A)** MiR-149-5p was pulled down and enriched with biotinylated circHIPK3 probe (n = 3; **p < 0.01). **(B)** The relative luciferase activity of circHIPK3 was markedly suppressed when co-transfected with wild circHIPK3 and miR-149-5p mimics compared with the control reporter or mutated luciferase reporter (n = 3; ***p < 0.001). **(C)** qRT–PCR detection showed that the expression of miR-149-5p was significantly increased/reduced in the circHIPK3 knockdown/overexpression group compared with each control group (n = 3; *p < 0.05, **p < 0.01). **(D)** The level of circHIPK3 could be significantly reduced/enhanced by miR-149-5p mimics/inhibitor compared with each control group (n = 3; *p < 0.05, **p < 0.01). **(E)** Luciferase reporter assays showed that the luciferase activity of FOXO1 wild-type reporter was significantly reduced when transfected with miR-149-5p mimics compared with control reporter or mutated luciferase reporter (n = 3; ***p < 0.001). **(F)** A significant enrichment of FOXO1 mRNA in the pulled down sediments of 3′ end biotin-labeled miR-149-5p. (n = 3; **p < 0.01). Results are expressed as the mean ± S.E.M. CTL = PBS control group. si-scrl = scramble siRNA. NC = negative control.

Accumulated studies have reported that the circRNA may serve as a competing endogenous RNA (ceRNA) or a molecular sponge to interact with miRNA (Chen, 2020). To further ascertain the characteristics of circHIPK3, we scanned circbase (http://www.circbase.org/), and the spliced sequence of circHIPK3 (hsa_circ_0000284) is shown in Supplementary Figure S3. Considering the miRNA sponge function of circHIPK3, we predicted five miRNAs (miR-149-5p, miR-515-5p, miR-637, miR-619, and miR-215) that interacted with circHIPK3 based on bioinformatics methods and RNA pull-down analysis using a circHIPK3 probe. The pull-down efficiency (Supplementary Figure S4A) and expressions of the five miRNAs detected with qRT–PCR were shown (Supplementary Figure S4B). Pull-down with the circHIPK3-specific probes which were oligonucleotides complementary to the back-splice junction of circHIPK3, but not the control probe, yielded a 11-fold enrichment of miR-149-5p in the pulled down sediments (Figure 2A). As to the five miRNAs, at least one miRNA-binding site of circHIPK3 defined by Arraystar proprietary algorithms was predicted with the help of the CircInteractome database (Supplementary Figure S4C). We then constructed a luciferase reporter plasmid containing the full length of circHIPK3. A luciferase assay that was performed to detect the binding of these five miRNAs showed that only miR-149-5p had strongly reduced the luciferase activity more than 50%, compared with the control (Supplementary Figure S4D, Figure2B). The efficiency of circHIPK3 knockdown/overexpression is shown in Supplementary Figure S2. The qRT–PCR analysis showed that circHIPK3 overexpression or knockdown could further reduce or increase the miRNA expression relative to levels in control RA-FLS, but miR-149-5p was the most significant one (Supplementary Figure S4E, Figure 2C). Moreover, experiments showed that the level of circHIPK3 could be significantly reduced/enhanced by miR-149-5p mimics/inhibitor compared with each control group (Figure 2D), indicating that circHIPK3 could bind miR-149-5p to regulate its level. The potential targets of miR-149-5p were investigated with the help of bioinformatics databases such as Targetscan and Starbase. A putative binding site was found in the 3’ UTR of the FOXO1 mRNA, which was a completely complementary sequence of the seed region of miR-149-5p (Supplementary Figure S4C). To confirm whether the FOXO1 could be regulated by miR-149-5p, we constructed luciferase reporters containing wild-type and mutated putative binding sites of FOXO1 transcripts. Luciferase reporter assays showed that the luciferase activity of the FOXO1 wild-type reporter was significantly reduced when transfected with miR-149-5p mimics, compared with the control reporter or mutated luciferase reporter (Figure 2E). The wild-type and mutated putative binding sites of circHIPK3 and FOXO1 with miR-149-5p are, respectively, shown in Supplementary Figure S5. Furthermore, we applied the biotinylated RNA pull-down assay to confirm the results. It was shown that the FOXO1 mRNA captured by biotin-miR-149-5p was found to be prominently enriched compared with the control group (Figure 2F).

### MiR-149-5p/FOXO1 Was Involved in CircHIPK3 Mediated VEGF Production of RA-FLS

**FIGURE 3:**
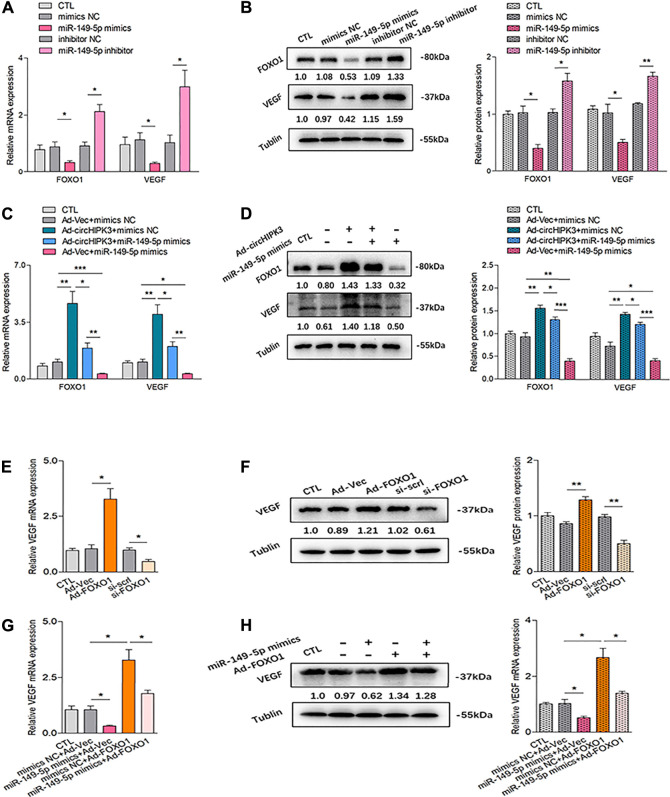
Confirmation of the circHIPK3/miR-149-5p/FOXO1/VEGF functional module *in vitro*. **(A–B)** FOXO1/VEGF mRNA **(A)** and protein expression **(B)** were obviously down-/up-regulated by miR-149-5p mimics/inhibitor (n = 3; *p < 0.05, **p < 0.01). **(C–D)** Co-effects of circHIPK3 and miR-149-5p on FOXO1/VEGF expression in RA-FLS were evaluated. FOXO1/VEGF mRNA **(C)** and protein. **(D)** The expressions were significantly elevated/reduced by circHIPK3 overexpression/miR-149-5p mimics compared with those of each control group. Moreover, the significant downregulation of FOXO1/VEGF expression repressed by miR-149-5p mimics could be obviously rescued by circHIPK3 overexpression; in other words, the significant upregulation of FOXO1/VEGF expression induced by circHIPK3 overexpression could be significantly inhibited by miR-149-5p mimics (n = 3; *p < 0.05, **p < 0.01, ***p < 0.001). **(E–F)** The mRNA **(E)** and protein **(F)** expression of VEGF in RA-FLS were significantly elevated/reduced by FOXO1 overexpression/knockdown (n = 3; *p < 0.05, **p < 0.01). **(G–H)** FOXO1 overexpression significantly upregulated VEGF mRNA **(G)** and protein **(H)** expression, and the increment of VEGF expression induced by FOXO1 overexpression could be significantly suppressed by miR-149-5p mimics (n = 3; *p < 0.05). Results are expressed as the mean ± S.E.M. CTL = PBS control group. si-scrl = scramble siRNA. NC = negative control.

Analyses showed that mRNA and protein levels of FOXO1 were significantly decreased by miR-149-5p mimics and increased by miR-149-5p inhibitor (Figures 3A,B). Moreover, the significant downregulation of FOXO1 expression repressed by miR-149-5p mimics could be significantly rescued by circHIPK3 overexpression; in other words, the significant upregulation of FOXO1 expression induced by circHIPK3 overexpression could be evidently inhibited by miR-149-5p mimics (Figures 3C,D). These results suggest that the circHIPK3 could regulate the FOXO1 expression *via* miR-149-5p.

The relationship between VEGF and circHIPK3/miR-149-5p/FOXO1 axis was further explored. We first explored the effect of FOXO1 on VEGF expression. Both overexpression and silencing of FOXO1 were constructed in RA-FLS, and the stable transfection efficiency of FOXO1 is, respectively, shown in Supplementary Figure S6. As demonstrated in Figures 3E,F, the overexpression of FOXO1 enhanced VEGF expression, while the silencing of FOXO1 presented a repressed effect on the VEGF level. Interestingly, the upregulation of VEGF expression induced by FOXO1 overexpression can be inhibited by miR-149-5p mimics, which could also significantly reduce the VEGF expression by itself (Figures 3G,H). Further experiments confirmed that the expression of VEGF tended to be consistent with the changing trend of FOXO1 expression (Figures 3C,D). As a matter of fact, these findings indicated that FOXO1 mediated the promotion of VEGF expression in RA-FLS, suggesting that VEGF is the downstream of circHIPK3/miR-149-5p/FOXO1 axis, constituting a functional module.

### CircHIPK3/miR-149-5p/FOXO1/VEGF Functional Module Was Involved in Antiangiogenic Effect of ATO *In Vitro*

**FIGURE 4:**
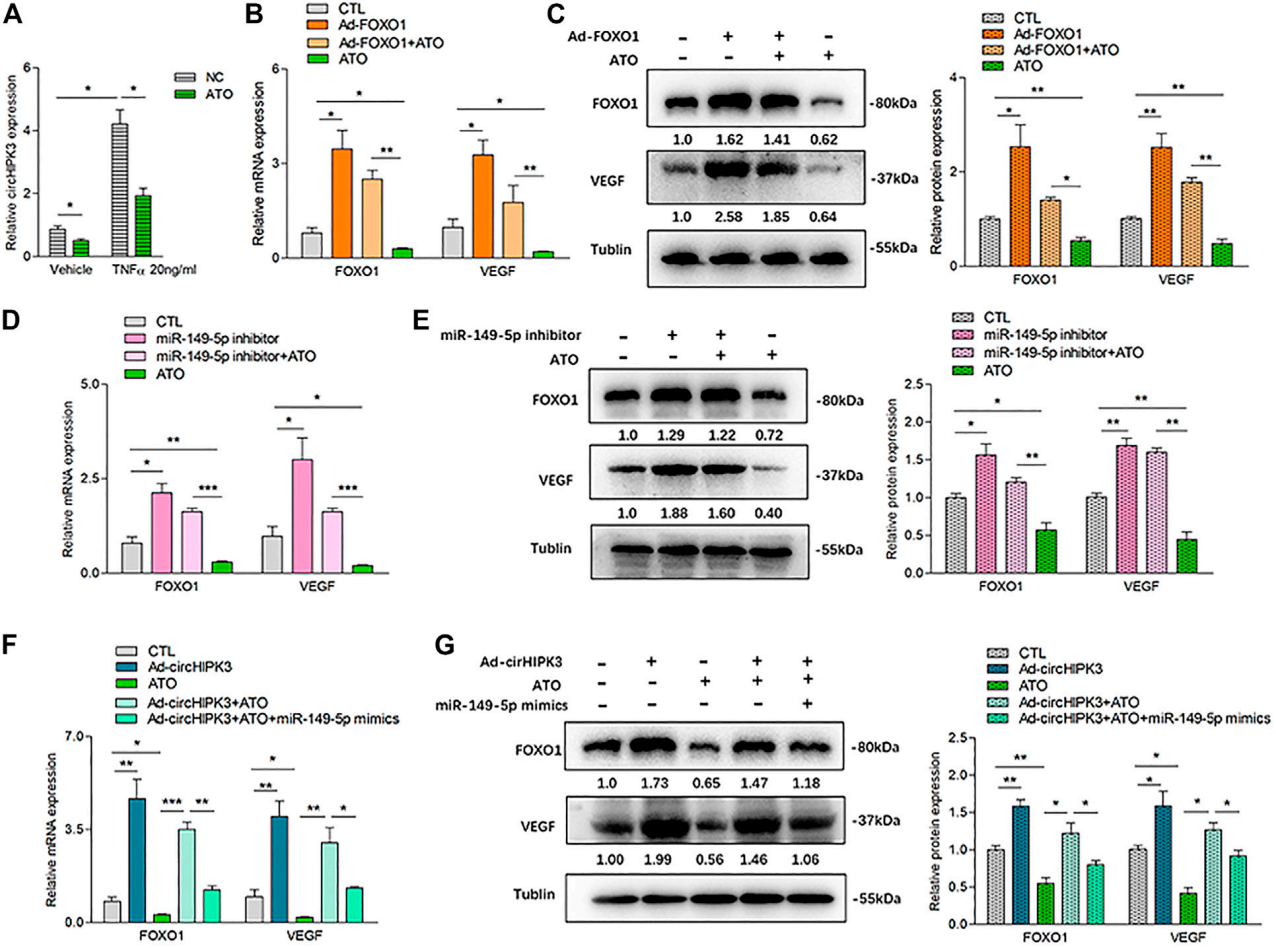
ATO inhibited angiogenesis *via* modulating the circHIPK3/miR-149-5p/FOXO1/VEGF functional module in RA-FLS *in vitro*. **(A)** After RA-FLS were exposed to 50 ng/ml TNF-α and 2.0 µM ATO for 48h, the expression of circHIPK3 was upregulated by TNF-α induction, and this upregulation could be significantly suppressed by ATO treatment (n = 3; *p < 0.05). **(B–C)** The expression of FOXO1/VEGF mRNA **(B)** and protein **(C)** were significantly upregulated by FOXO1 overexpression and downregulated by ATO. Furthermore, the FOXO1/VEGF downregulation induced by ATO could be rescued partially by FOXO1 overexpression (n = 3; *p < 0.05, **p < 0.01). **(D–E)** The expression of FOXO1/VEGF mRNA **(D)** and protein **(E)** were significantly upregulated by miR-149-5p inhibitor, and downregulated by ATO. Additionally, the FOXO1/VEGF expression downregulated by ATO could be partially rescued by miR-149-5p inhibitor (n = 3; *p < 0.05, **p < 0.01, ***p < 0.001). **(F–G)** The expression of FOXO1/VEGF mRNA **(F)** and protein **(G)** were significantly upregulated by circHIPK3 overexpression, and downregulated by ATO. Moreover, the expression of FOXO1/VEGF downregulated by ATO could be partially rescued by circHIPK3 overexpression but significantly reduced again further upon miR-149-5p mimics addition (n = 3; *p < 0.05, **p < 0.01, ***p < 0.001). Results are expressed as the mean ± S.E.M. CTL = PBS control group.

In our previous study, we found that ATO could inhibit VEGF expression in RA-FLS. Therefore, we wondered whether ATO could play an antiangiogenic role *via* the inhibition of circHIPK3. The results demonstrated that the level of circHIPK3 was decreased by ATO after RA-FLS was exposed to 2.0 µM ATO for 48 h, and the downregulation of circHIPK3 was more significant under TNF-α induction (Figure 4A). To further verify the intervention of the circHIPK3/miR-149-5p/FOXO1/VEGF functional module in the effect of ATO’s antiangiogenic effect, at the same time of TNF-α induction, we performed experiments of FOXO1 overexpression, miR-149-5p inhibition, and circHIPK3 overexpression, and the simultaneous treatment of ATO, followed by the detection of FOXO1 and VEGF expression. Exposure of RA-FLS to ATO induced decrement of FOXO1/VEGF mRNA and protein levels, and intriguingly, this decrement could be rescued partially by FOXO1 overexpression (Figures 4B,C), miR-149-5p inhibitor (Figures 4D,E), or circHIPK3 overexpression (Figures 4F,G). Apart from that, FOXO1/VEGF levels downregulated by ATO, further partially rescued by circHIPK3 overexpression, however significantly reduced again further upon miR-149-5p mimics addition (Figures 4F,G). These results verified that ATO could exhibit the antiangiogenic effect through regulating circHIPK3/miR-149-5p/FOXO1-mediated VEGF expression.

### AAV-Mediated circHIPK3 Knockdown or Its Combined Application With ATO Mitigated the Severity of Arthritis in CIA Mice

**FIGURE 5:**
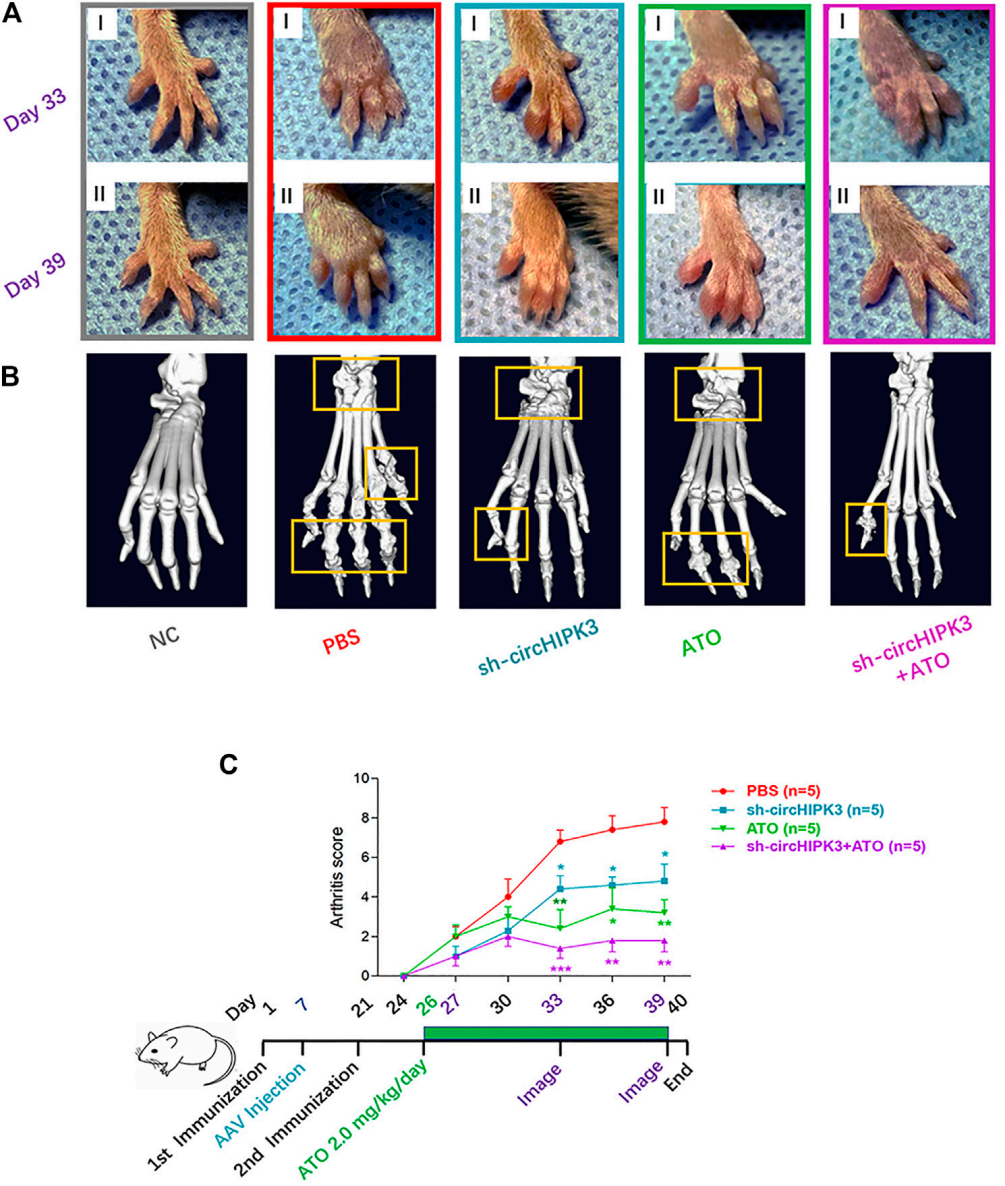
CircHIPK3 knockdown or its combined application with ATO alleviated the symptoms and joint destruction of CIA mice. **(A)** Representative pictures of the paws of mice showed that CIA developed rapidly in paws after the mice were immunized with collagen II and sustained to the end of the experiment. AAV-sh-circHIPK3, 2.0 mg/kg/day ATO administration, or the combination treatment of above two agents significantly alleviated the severity of arthritis compared with the CIA group on day 33 (Ⅰ) and day 39 (Ⅱ) after the first immunization. **(B)** The micro-CT images of paws demonstrated the destruction of the bone and cartilage in CIA mice, and ATO injection alleviated the joint destruction. AAV-sh-circHIPK3 intra-articular therapy in addition to ATO could evidently reduce the damages of bone compared with ATO therapy alone. **(C)** Clinical evaluation and the process diagram are demonstrated. Clinical arthritis scores showed that both ATO and intra-articular AAV-sh-circHIPK3 therapy could significantly reduce the arthritis scores of CIA mice. Moreover, combining ATO and AAV-sh-circHIPK3 reduced the arthritis score more significantly than ATO treatment alone (n = 5; *p < 0.05, **p < 0.01, ***p < 0.001 versus PBS group). Data are expressed as the mean ± SEM.

Our previous study confirmed that ATO has a significant antiangiogenic effect on the CIA synovium *via* its suppression on the VEGF angiogenic functional module (Zhang et al., 2017). To check if the inhibitory effect of ATO on the VEGF module is partially due to the effect of ATO on circHIPK3, and to verify the anti-circHIPK3/miR-149-5p/FOXO1/VEGF effect of ATO could be replicated *in vivo*, the collagen II–induced arthritis (CIA) model was established, and AAV-sh-circHIPK3 or AAV-sh-CMV-EGFP viral vectors as a control were intra-articularly administered to CIA mice on the seventh day after first (primary) immunization. AAV effectively transduced into synovial tissue and displayed a local distribution pattern *in vivo* (Supplementary Figure S7A). We assessed the severity of arthritis from day 24 after primary immunization according to a standard arthritic score system (Zhang et al., 2017). CIA developed rapidly in paws after mice were immunized with collagen II, and showed evident clinical symptoms sustainably (Figures 5A,C). AAV-sh-circHIPK3 significantly alleviated the severity of arthritis and reduced arthritic scores compared with the CIA control group (Figures 5A,C), whereas the control AAV-shRNA treatment had no significant effect on CIA arthritis (Supplementary Figure S7B). Then, the curative effect of ATO treatment plus AAV-sh-circHIPK3 injection was evaluated. ATO treatment at a dose of 2.0 mg/kg/day per mouse lasted from day 26 to day 39 after the first immunization. As shown in Figures 5A,C, ATO exhibited continuously significant suppression from day 26 to day 39. Intriguingly, the effect of ATO treatment in the CIA mice could be enhanced by AAV-sh-circHIPK3 injection (Figures 5A,C), while the control AAV-shRNA injection had no significant effect on the ATO’s curative effect (Supplementary Figure S7B). Process diagrams are demonstrated in Figure 5C. The evaluation of ATO and AAV-sh-circHIPK3 injection toxicity were performed as described before (Zhang et al., 2017), and no side effects, such as diarrhea, behavioral and hair abnormalities, or abnormal death, were observed during the study. Body-weight loss was also recorded to evaluate the curative effects and the toxicity of ATO and AAV-sh-circ-HIPK3 injection. PBS-treated CIA mice showed a significant decrease in weight compared with the normal mice from day 24 to day 39. ATO as well as the combination therapy of ATO and AAV-sh-circ-HIPK3 injection significantly increased body weight compared with the CIA control group. Furthermore, there was no significant difference of body weight between the combination therapy group and normal mice group (Supplementary Figure S7C).

**FIGURE 6:**
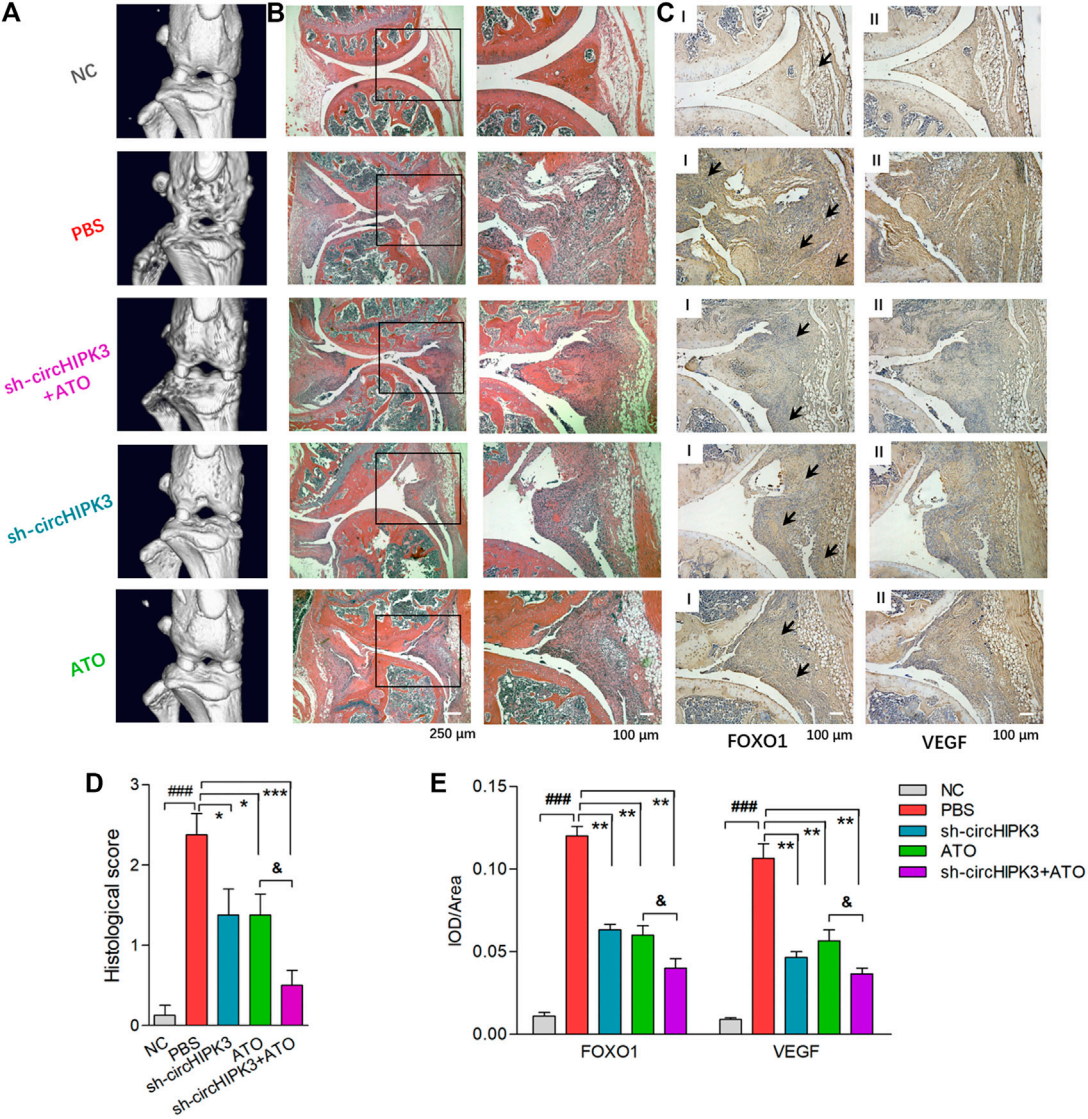
Histological evaluation of single or combined treatment of AAV-sh-circ-HIPK3 and ATO. **(A)** Micro-CT images of knee joints demonstrated the destruction of bone and cartilage in CIA mice, and ATO injection alleviated the joint destruction. AAV-sh-circHIPK3 combined with ATO could significantly reduce the damages of bone compared to ATO therapy alone. **(B)** The severity of arthritis in CIA mice was assessed by H&E staining of knee joint sections. ATO treatment at 2.0 mg/kg/day and AAV-sh-circHIPK3 injection, respectively, significantly reduced synovial hyperplasia, cartilage/bone erosion and joint inflammation compared with CIA control mice. Additionally, the combination therapy of ATO and AAV-sh-circHIPK3 yielded more reduction of pannus formation and inflammatory infiltration. **(C)** Immunohistochemical analysis was performed to evaluate the effect of AAV-sh-circHIPK3 and ATO on FOXO1 (Ⅰ) and VEGF (Ⅱ) expression in synovial tissues of CIA mice. CIA mice under PBS treatment had significantly increased staining intensity of FOXO1 and VEGF compared with normal mice. And the injection of AAV-sh-circHIPK3 or ATO significantly decreased staining for FOXO1 and VEGF compared with CIA control mice. Interestingly, FOXO1 and VEGF staining in the combined treatment of AAV-sh-circ-HIPK3 and ATO group were significantly weaker than those in the CIA mice under ATO therapy alone. Histological scores **(D)** and average IOD values for FOXO1 and VEGF immunostaining in the knee joint synovium **(E)** were, respectively, evaluated and shown (n = 5; *p < 0.05, **p < 0.01, ***p < 0.001 versus PBS treatment group; ###*p* < 0.001 versus NC; and *p* < 0.05 versus ATO group). Data are expressed as the mean ± SEM. NC = normal control group.

Consistent with the trend toward clinical symptoms, a three-dimensional reconstruction of micro-CT images demonstrated the destruction of bone and cartilage in the paws and knees from CIA mice, and AAV-sh-circHIPK3 intra-articular injection or ATO treatment significantly rescued the destructed joint structure. And joint destruction in the combination group of AAV-sh-circHIPK3 and ATO was more significantly inhibited than AAV-sh-circHIPK3 or ATO therapy alone (Figure 5B, Figure 6A). The mean CT values of the paw and knee joints are evaluated and shown, respectively, in Supplementary Figure S7D–E.

The severity of arthritis in the CIA mice was further evaluated *via* histological analysis (hematoxylin and eosin stain, i.e., H&E staining) of knee joint sections. Synovial hyperplasia, pannus formation, and massive joint destruction with the erosion of cartilage were observed in the knee joints of the CIA mice, whereas no synovial hyperplasia or pannus formation was found in normal mice. There was significant decreased inflammation, and cartilage and bone destruction in mice after the injection of AAV-sh-circHIPK3 or ATO. More interestingly, bone destruction and inflammatory cell infiltration were significantly improved in the combinatory treatment group of AAV-sh-circHIPK3 and ATO compared with ATO therapy alone (Figures 6B,D).

### AAV-Sh-circHIPK3 Combined With ATO Treatment Suppressed the circHIPK3/Mi-149-5p/FOXO1/VEGF Functional Module in Synovial Tissue of CIA Mice

qRT–PCR were performed to evaluate the expression levels of circHIPK3 and miR-149-5p in the synovium of different groups (Supplementary Figure S8). Synovial circHIPK3/mi-149-5p levels in CIA mice treated with PBS were significantly higher/lower than those in normal mice. Moreover, circHIPK3/mi-149-5p levels in CIA mice could be significantly decreased/increased by AAV-sh-circHIPK3, ATO at a dose of 2.0 mg/kg/day, or AAV-sh-circHIPK3+ATO combination treatment. Most notably, circHIPK3/mi-149-5p levels in CIA mice treated with AAV-sh-circHIPK3+ATO combination therapy were significantly lower/higher than those in CIA mice treated with ATO single therapy.

Immunohistochemical analysis was performed, and the average integrated optical density (IOD) values were calculated to evaluate the effect of AAV-sh-circHIPK3 on the FOXO1 (Figure 6C) and VEGF (Figure 6C) expression in the synovial tissue of CIA mice. Normal mice showed weak immunostaining of FOXO1 and VEGF, while CIA mice under PBS treatment had significantly increased the staining intensity for FOXO1 and VEGF in knee joint synovium, and furthermore, the staining intensity for FOXO1 and VEGF in the CIA mice were significantly decreased by AAV-sh-circHIPK3–injected treatment (Figures 6C,E). On the other hand, the immunostaining intensity for FOXO1 and VEGF were significantly weaker under ATO treatment at a dosage of 2.0 mg/kg/day than those of CIA mice treated with PBS (Figures 6C,E). Interestingly, the immunostaining intensity for FOXO1 and VEGF in the synovium from CIA mice treated with injection of AAV-sh-circHIPK3 combined with ATO were significantly weaker than those of CIA mice treated with ATO alone (Figures 6C,E). In other words, the AAV-sh-circHIPK3 injection enhanced ATO-induced decrement of FOXO1 and VEGF levels in synovial tissue of CIA mice. Together, our results indicated the formation of the circHIPK3/mi-149-5p/FOXO1/VEGF functional module in the synovial tissue of CIA mice, and circHIPK3 suppression is partly involved in the inhibitory effect of ATO on VEGF expression.

## Discussion

Our present study focused on the effect of circHIPK3 which was remarkably elevated by inflammation induction in RA-FLS and then influenced VEGF secretion with its induced increment of synovial vascularity. Further knockdown of circHIPK3 inhibited the VEGF production and thus the RA-FLS induced angiogenesis, which indicated that the circHIPK3 functioned as a pathological angiogenesis-associated gene in RA. CircHIPK3 is highly expressed in many tissues, mainly originating from the second exon of the gene HIPK3. The recent progress of the mechanisms and functions of circHIPK3 were mainly focused in the fields of malignancies (Zhang et al., 2020). Until recently, the circHIPK3 has been found to play essential roles on endothelial cell function and vessel growth in vascular diseases such as pulmonary arterial hypertension (PAH) (Hong et al., 2021), and this angiogenic effect of circHIPK3 was in accordance with that proposed by Xiaoyun (Si et al., 2020). However, this is the first time the pro-angiogenic effect of circHIPK3 under inflammatory microenvironment is reported in our study. Since there is a vicious circle of mutual promotion between inflammation and angiogenesis in various diseases (Capitão and Soares, 2016; Whiteford et al., 2016) besides diabetic retinopathy, tumor growth, atherosclerosis and arthritis, the discovery of key nodes in this complicated relationship will be of great significance to the development of treatment. And from this study, circHIPK3 was supposed to play a core role in abnormal angiogenesis under inflammatory microenvironment and serve as a key target for therapy.

In addition, our findings verified the binding sites between circHIPK3 and miR-149-5p, indicating that circHIPK3 might act as an miR-149-5p sponge to modulate its function in RA-FLS. Our present study uncovered that miR-149-5p mimics inhibited the VEGF expression, while miR-149-5p inhibitor upregulated the VEGF expression in RA-FLS, indicating that miR-149-5p acted as an angiogenic suppressor in RA-FLS, and the positive effect of miR-149-5p toward the alleviation of the disease in inflammatory condition was consistent with the previous study (Wang et al., 2020) in the chondrocytes of osteoarthritis. By using bioinformatics, FOXO1 was predicted to be the target of miR-149-5p and further testified. As to the results of different target genes of miR-149-5p, tissue variability or complicated local context might give the explanation, and full investigation still needs to be undertaken in the future.

One of the most common functional mechanisms of miRNAs is the inhibition of miRNAs on gene expression at the transcriptional or posttranscriptional levels by binding to the 3′UTR of target mRNA. And further study illuminated that FOXO1 was the target of miR-149-5p. The FOXO family of forkhead transcription factors is at the crossroads of many signal transduction pathways that are evolutionarily conserved. FOXO1, a main isoform of FOXO, has emerged as an important regulator of angiogenesis at different levels (Kousteni, 2011). In this study, FOXO1 overexpression/silencing plus miR-149-5p inhibitor/mimics treatment affected FOXO1 levels, with similar changing trends of VEGF expression. Moreover, the upregulation of VEGF expression induced by the FOXO1 overexpression could be inhibited by miR-149-5p mimics. The above results indicated that FOXO1, targeted by miR-149-5p, could promote the TNF-α–induced VEGF expression of RA-FLS and thus angiogenic responses. Therefore, the positive relationship of FOXO1 with VEGF expression suggests that FOXO1 was the upstream of VEGF modulation, and FOXO1 might act as a pathogenic gene in RA synovial tissues. The result of promotional effects of FOXO1 on angiogenesis was consistent with the results of studies performed in keratinocytes (Jeon et al., 2018) and chondrocytes (Zhang et al., 2019b) required for angiogenesis in wound healing. Stages of disease development will relate to the different manifestations of FOXO1’s effect on disease progression. The promotion of angiogenesis in the early stage might be beneficial to tissue repair, while the overexpression of FOXO1 in the later stage might be harmful. Besides, in this study, five miRNA candidates (miR-149-5p, miR-515-5p, miR-637, miR-619, and miR-215) which might interact with circHIPK3, were predicted and screened. And miR-149-5p was confirmed to be most likely to bind to circHIPK3. However, it has also been reported that circHIPK3-miR-637-STAT3 could promote oxaliplatin resistance in colorectal cancer through autophagy (Zhang et al., 2019c), and circHIPK3-miR-215-5p-YY1 had the ability to regulate melanoma cell behaviors (Zhu and Sun, 2020). Multiple interactions of one circRNA with miRNAs–mRNAs is not uncommon, and this might be related to the specificity of species or local tissues relevant with circRNA’s function, worthy of future in-depth studies.

**FIGURE 7:**
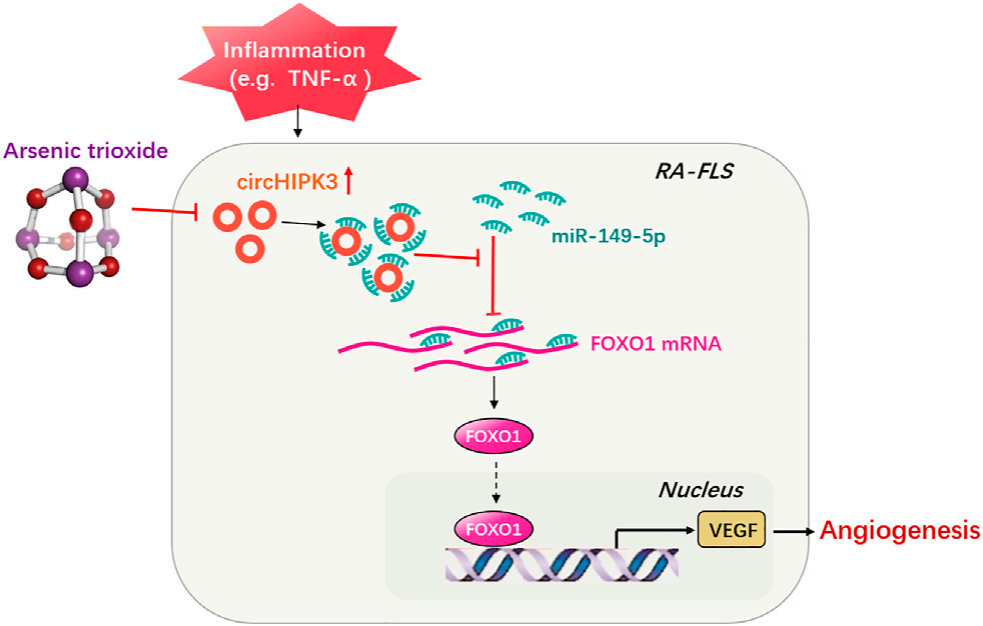
Schematic illustration underlying the mechanism of ATO’s effect on the circHIPK3/miR-149-5p/FOXO1/VEGF functional module. CircHIPK3 might act as a sponge for the FOXO1-targeting miR-149-5p, leading to the downregulation of downstream VEGF. These networked factors mainly form a functional module regulating angiogenesis in RA-FLS, and the expression of this functional module could be significantly downregulated by ATO, ultimately inhibiting angiogenesis.

More importantly, in this study, ATO was found to suppress the VEGF activity through reducing circHIPK3 overexpression in RA-FLS, especially under inflammatory factor TNF-α′s induction. To verify the specificity of ATO’s targeting, FOXO1 overexpression and miR-149-5p inhibitor were performed on the basis of ATO treatment. Results showed that the FOXO1 and VEGF expression were significantly decreased by ATO, and moreover, FOXO1/VEGF downregulation induced by ATO could be partially rescued by the aforementioned FOXO1 overexpression or miR-149-5p inhibitor intervention. Furthermore, FOXO1/VEGF levels downregulated by ATO could be rescued partially by circHIPK3 overexpression but significantly reduced again further upon miR-149-5p mimics addition, indicating the circHIPK3/miR-149-5p/FOXO1 pathway was involved in the inhibitory effect of ATO on VEGF (Figure 7). We have previously reported that ATO could inhibit the RA synovial angiogenesis *via* the VEGF functional module; therefore, the results of the present study undoubtedly provided an in-depth insight into the antiangiogenic activities of ATO, revealing a new level of diversity in the regulatory mechanisms of ATO therapy on diseases. So far, there is lack of any report about ATO’s treatment of diseases through circRNA’s relevant mechanisms, since the acknowledgment of the relationship between circRNAs (or other noncoding RNAs such as lncRNA) and ATO, even other chemical drugs, is still limited to toxicology (Dai et al., 2018; Xiao et al., 2018) and drug resistance in the field cancer (Li et al., 2020). The different effects of ATO on human health might be associated with the complexity of tissue microenvironment with various inflammatory backgrounds, or even related to the genetic and epigenetic mechanisms. The exposure of arsenic may perturb DNA methylation patterns and subsequently alter the expression of key genes, while consensus m^6^A motifs are enriched in circRNAs (Yang et al., 2017); therefore, arsenic might influence the expression of certain circRNAs *via* its effect on m^6^A motifs of circRNAs or their derived genes. On the other hand, arsenic-induced histone modifications have been identified (Zhang et al., 2019d), and epigenetic changes in histones and genomes may affect alternative splicing and directly affect the biogenesis of circRNAs (Kristensen et al., 2018). Quite rightly, further exact mechanisms are yet to be investigated. The results of this study could provide a valuable basis for evaluating the molecular mechanisms of ATO on diseases with full objectivity and comprehensiveness. This favorable antiangiogenic profile of ATO is important because it enables us to formulate more rational drug applications that could exert therapeutic effect. The chronic toxicity with long-term use of ATO is indeed an important reason which restricts the clinical transformation of ATO for RA treatment, and it is also an important reason for our series of research works on ATO, such as autophagy (Wang et al., 2019), immunity (Li et al., 2019), angiogenesis (Zhang et al., 2017), and even circRNA in this study, because only the continuous in-depth understanding of ATO can help to “make best use of the advantages and bypass the disadvantages.” We even hope that one day, targeted treatment of ATO could be applied in different subtypes of disease, or ATO’s combination treatment with other drugs of varied mechanisms, achieving the purpose of reducing toxicity and improving effect ultimately. As circHIPK3 has also been verified to be a vital pathogenic gene in many cancers (Zhang et al., 2020), and inflammatory angiogenesis is a key pathogenetic sector as well, plus the mitigation of arthritis in our study was turned out to be more evident upon the application of both ATO and circHIPK3 silencing, combination therapy of circHIPK3 knockdown with ATO may be a potential therapeutic strategy for the future treatment.

While closing, our study observed that circHIPK3 upregulated the VEGF-induced angiogenesis of the synovium by targeting miR-149-5p/FOXP1 in RA-FLS under inflammatory induction for the first time, contributing to provide potential targets for RA precision therapy. Moreover, ATO may have an antiangiogenic effect on RA-FLS and the synovium in CIA mice through this circHIPK3/miR-149-5p/FOXP1/VEGF functional module, indicating that the combination treatment of circHIPK3 silencing with ATO may be a potential therapeutic strategy for RA and even malignancies.

## Materials and Methods

### Cell Cultures and Reagents

RA fibroblast–like synoviocytes (RA-FLS) were purchased from Cell Applications (San Diego, CA, United States), and human dermal microvascular endothelial cells (HDMECs) were obtained from ScienCell Research Laboratories (6076 Corte Del Cedro, Carlsbad, CA, United States). Cells at passages three to six were used for experiments. The co-culture system of RA-FLS and HDMECs was established as described previously (Zhang et al., 2017). Briefly, RA-FLS (4×10^5^ cells/ml) and HDMECs (ranging from 2×10^5^ cells/ml to 3×10^5^ cells/ml) were seeded in the lower and upper chambers of a transwell apparatus (Costar), respectively, and incubated in 2.0 μM arsenic trioxide (ATO; As_2_O_3_; Yitaida Pharmaceutical Factory, Harbin, Heilongjiang, China) with or without TNF-α (50 ng/ml, Peprotech, Rocky Hill, United States). After being cultured in 1% FBS DMEM for 48 h, the supernatants were harvested. Fresh supernatants were taken out for the subsequent transwell assays, tube formation tests, and *ex vivo* aortic ring angiogenesis assays. Remaining supernatants were frozen at -20°C until analysis by commercial enzyme-linked immunosorbent assay (ELISA) kit for VEGF (DVE00, R&D Systems). The mRNA and protein expression in RA-FLS co-cultured were, respectively, determined by quantitative real-time PCR (qRT-PCR) and Western blot analysis.

### HDMEC Transwell and Tube Formation Assays *In Vitro*, *Plus* Mouse Aortic Ring Angiogenesis Assays *Ex Vivo*

Transwell and mouse aortic ring angiogenesis assays were performed as described previously (Zhang et al., 2017). Briefly, as to transwell assays, the lower chamber was filled with 600 µl of supernatants from the cocultures. HDMECs (3×10^4^ cells) were added to each upper chamber and incubated for 6 h. Cells that migrated to the lower phase were stained and quantified. Aortic rings from thoracic aortas of 8-week-old C57BL/6 mice (SPF, Laboratory Animal Center of the Second Affiliated Hospital of Harbin Medical University) were incubated in 1% FBS EGM-2 on the basis of Matrigel. After verifying sprouts from the aortic rings, the EGM-2 was replaced with 500 μl of fresh supernatants from cocultures, and the aortic rings were further incubated for 3 days. Microvessel outgrowths were examined and analyzed using ImageJ software. In tube formation assays, a µ-Slide (ibidi, GmbH, Munich, German) was used to investigate angiogenesis. 10 μl Matrigel (Corning) was plated per inner well, and 70 μl of cell suspension containing 1×10^4^ cells was added to each upper well for a further 6-h incubation. All images were obtained using microscopy (LEICA DMi8).

### RNA Preparation and qRT-PCR

Total cellular and tissue RNA was isolated by Trizol (Invitrogen) (Chu and Zhang, 2018). As to RNA preparation for circRNA determination, total RNA (5 µg) was first treated with RNase R (Geneseed Biotech, Guangzhou, China), and GAPDH in the same sample without RNase R treatment was used as an internal control. For the detection of circRNA and mRNA, RNA and PrimeScript RT Master Mix (Takara Biotechnology, Dalian, China) were used in reverse transcription. The qRT–PCR amplification was performed using the iTaq Universal SYBR Green supermix (Bio-Rad). For cellular and tissue microRNA, total RNA was extracted by using the QIAzol Lysis Reagent. Reverse transcription and qRT-PCR were performed with riboSCRIPTTM Reverse Transcription Kit (RiboBio, Guangzhou, China) and Applied Biosystems TaqMan MicroRNA Assay Kit. Relative circRNA, mRNA, or miRNA expression was normalized to GAPDH or U6 snRNA levels using the 2^−ΔΔCt^ method, respectively. The sequence for each primer is listed in Supplementary Table S1.

### Fluorescence *In Situ* Hybridization Assay (FISH)

FISH assays were performed with Fluorescent *In situ* Hybridization Kits (Ribobio, Guangzhou, China). Briefly, RA-FLS grown on coverslips were fixed with 4% paraformaldehyde at room temperature for 10 min and treated with 0.5% Triton X-100 at 4°C for 5 min. The samples were treated with a pre-hybridization buffer at 37°C for 30 min and then in hybridization buffer at 37°C for 12–16 h with a CY3-labeled circRNA probe (Ribobio, Guangzhou, China, Supplementary Table S1) in a humid and dark environment. The samples were mounted with a fluorescence mounting medium and imaged by the use of a laser confocal microscope (TCS SP8 X, LEICA).

### Bioinformatics Analysis

The miRNA target of circHIPK3 was predicted by bioinformatics databases, namely, Targetscan (http://www.targetscan.org/), CircInteractome (https://circinteractome.nia.nih.gov/), and Rnahybrid (https://bibiserv.cebitec.uni-bielefeld.de/rnahybrid/). The mRNA target of miR-149-5p was predicted by bioinformatics databases such as Targetscan (http://www. targetscan. org/) and Starbase (http://starbase.sysu.edu. cn/).

### Virus Infection and Cell Transfection

Adenovirus vector encoding circHIPK3 (Ad-circHIPK3), FOXO1 (Ad-FOXO1), and their GFP control (Ad-Vector) were entrusted to Hanbio Biotechnology (Shanghai, China). In brief, circ HIPK3 and FOXO1 fragments were amplified and ligated with the linearized vector, and the amplified sequence was detected by Sanger sequencing to verify the consistency with circ HIPK3 or FOXO1. Packaging plasmids and viral vectors were co-transfected into HEK-293 T cells using Lipofectamine 3,000 transfection reagent (ThermoFisher). 48 h after transfection, the culture medium was centrifuged (SolarBio). Finally, the culture medium mixture with polybrene was added to RA-FLS.

SiRNAs that target human circHIPK3 (si-circHIPK3) and FOXO1 (si-FOXO1), the vectors that upregulate or downregulate relative miR-149-5p expressions (miR-149-5p mimic and miR-149-5p inhibitor), were designed and constructed by RiboBio (Guangzhou, China). Lipofectamine RNAiMAX (ThermoFisher) was further applied according to the manufacturer’s instructions. Relative sequences are shown in Supplementary Table S1.

### RNA Pull-Down Assay

As to biotin-coupled miRNA capture, the 3′end biotinylated miR-149-5p mimics or control RNA (Ribio, Guangzhou, China) were transfected into RA-FLS at a final concentration of 50 nM for 48 h before harvest. Then the lysis buffer [0.02 M Tris-HCl (pH 7.5), 0.1 M NaCl, 0.5 mM DTT, 0.5% NP-40, 0.6 U/µl RNase inhibitor (Promega), and complete protease inhibitor cocktail (Sigma, United States) were added into the cell pellets. Then the cell lysates with biotinylated probes were incubated with streptavidin-conjugated magnetic beads (Invitrogen, United States), the beads were washed for five times, and TRIzol reagent (Invitrogen, Thermo, United States) was added to the washed beads for RNA extraction. Then the abundance of circHIPK3 and FOXO1 mRNA in bound fraction pulled down were evaluated by qRT–PCR analysis. The biotinylated circHIPK3 probe was designed and synthesized by RiboBIO (Guangzhou, China). And the RNA pull-down assay was performed using an RNA pull-down kit (BersinBio, Guangzhou, China) according to the manufacturer’s instructions similar to the aforementioned biotin-coupled miRNA capture.

### Luciferase Reporter Assay

The expression plasmid of circHIPK3 was created by the placement of the human entire circHIPK3 sequence into pcDNA3.1 circRNA Mini Vector (Addgene). RA-FLS were transfected with an miR-149-5p mimic (RiboBio, China) or control mimic combined with luciferase reporter or an empty vector, and cells were also transfected with a pcDNA3.1-circHIPK3 and its mutant type using Lipofectamine 2000 (Invitrogen) according to the manufacturer’s protocol.

The reporter plasmid pGL3-FOXO1 containing the predicted miR-149-5p targeting regions was designed by Ribobio (China). A part of the wild-type and mutated 3′-UTR of FOXO1 was cloned immediately to the downstream of the firefly luciferase reporter. MiR-149-5p mimics and its negative control were transfected into RA-FLS at a final concentration of 100 nM using a riboFECTTM CP Transfection Kit (RiboBio, China) according to the manufacturer’s instructions. The luciferase activity was measured using Dual–Glo luciferase Assay System (Promega) with Cytation five Cell Imaging Multi-Mode Reader (BioTek) 24 h after transfection.

### Western Blot Analysis

Protein extraction and Western blot analysis were performed as described before (Chu and Zhang, 2018). The antibodies against specific proteins including VEGF (Abcam, ab214424) and FOXO1 (Cell Signaling Technology, 2880) were then added. GAPDH was utilized for normalization.

### Animal Model and Experimental Protocol

Experimental procedures were carried out in accordance with the Guide for the Care and Use of Laboratory Animals and were approved by the Institutional Animal Care and Use Committee of the First Affiliated Hospital of Harbin Medical University. The adeno-associated virus 5 (AAV5) of sh-circHIPK3 was constructed and packaged by HanBio (Shanghai, China), and the AAV5 containing shRNA control was provided at the same time. The collagen-induced arthritis (CIA) mouse model was established using 8-week-old male DBA/1J mice (19 ± 2 g in weight, SLAC, Shanghai, China) as described previously (Zhang et al., 2017). On day 7 after the first immunization, mice were randomly assigned to seven different groups (n = 5 per group): normal control group (mice without immunization and injected with PBS), CIA control group (CIA mice treated with PBS), AAV-sh-circHIPK3 treatment group, control shRNA group, ATO-treated group, AAV-sh-circHIPK3 + ATO group, and control shRNA + ATO group. In the AAV treatment groups, a total of 10 μl (approximately 3.0 × 10^12^ vg/ml) of AAV expressing sh-circHIPK3 or control shRNA were injected into the knee joint. To verify the successful AAV infection in the joint cavity, we used the joint frozen section to detect immunofluorescence of AAV5-CMV-EGFP on day 28 after virus injection. The mice were given intraperitoneal injections of ATO at a dose of 2.0 mg/kg/day from day 26 to day 39. The mice were euthanized on day 40, and a part of the synovial tissues were isolated from knee joints of mice for the subsequent tissue qRT–PCR test. A time line for ATO and AAV treatment is provided in Figure 5C. The body weight of each mouse was recorded every 3 days from day 24.

### Assessment of Arthritis Severity and Micro-CT Imaging

Clinical arthritis was assessed daily, and the arthritic score was evaluated every 3 days from day 24 after primary immunization to the end of the experiment period according to a scoring system (Zhang et al., 2017). Mice were scanned and reconstructed into a three-dimensional (3D) structure *via* micro-CT imaging (Quantum GX, Perkin Elmer, Waltham, United States) to evaluate joint bone damages on day 40 after the initial collagen injection. The mean CT values of the hind paws were calculated with Caliper Analyze software (Analyze Direct, Kansas, United States) to assess bone loss (Wang et al., 2019).

### Hematoxylin–Eosin Staining and Immunohistochemical Analysis

After being sacrificed, the knee joints of mice were collected and stained with HE staining. To quantitatively evaluate the severity of arthritis, a scoring system was employed referring to the reported protocol (Zhang et al., 2017). Histological changes were examined by microscopic evaluation and scored in a blinded manner by two independent observers.

The immunohistochemical analysis was performed as previously described with some modifications (Zhang et al., 2017). Knee joint sections on slides were incubated with anti-FOXO1 and anti-VEGF antibodies (Wanleibio, Shenyang, China). Subsequently, the sections were stained using the polymer HRP detection system (PV9001, ZSGB-BIO, Beijing, China) and were visualized with DAB Peroxidase Substrate Kit (ZLI-9017, ZSGB-BIO, Beijing, China). Following immunostaining, the synovium area in the joint of each section was evaluated under a microscope (LEICA DMi8) in three randomly selected areas at a magnification of 100×. Image-Pro Plus 6 (Media Cybernetics, Inc.) was used to analyze the average integrated optical density (IOD) according to a previously described protocol (Wang et al., 2019).

### Statistical Analysis

Data were presented as means ± S.E.M. Comparisons of means between two groups were performed *via* Student’s t-test. Comparisons of means among multiple groups were accomplished by one-way analysis of variance (ANOVA), and a multiple range least significant difference (LSD) was used for intergroup comparisons. *p* values < 0.05 were considered to be statistically significant. All statistical analyses were performed with SPSS 17.0 software.

## Data Availability

The raw data supporting the conclusions of this article will be made available by the authors, without undue reservation.
