# Supplementary material for: Angiogenesis is Inhibited by Arsenic Trioxide Through Downregulation of the CircHIPK3/miR-149-5p/FOXO1/VEGF Functional Module in Rheumatoid Arthritis
Source: Front Pharmacol. 2021 Oct 27;12:751667. doi: 10.3389/fphar.2021.751667 (PMC8579003; doi:10.3389/fphar.2021.751667)
Supplement: Supplementary file 1 [file DataSheet1.pdf]

Supplementary Figure 1

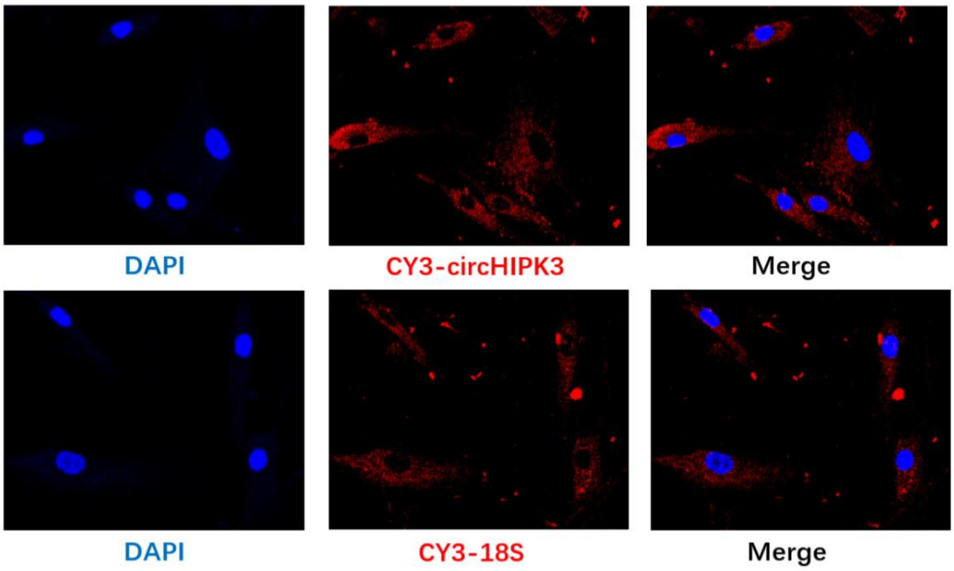

Supplementary Figure 1. Confirmation of circHIPK3 located in the cytoplasm.

FISH demonstrated the main location of circHIPK3 in the cytoplasm (n=3; red, circHIPK3; blue, DAPI nuclear staining).

Supplementary Figure 2

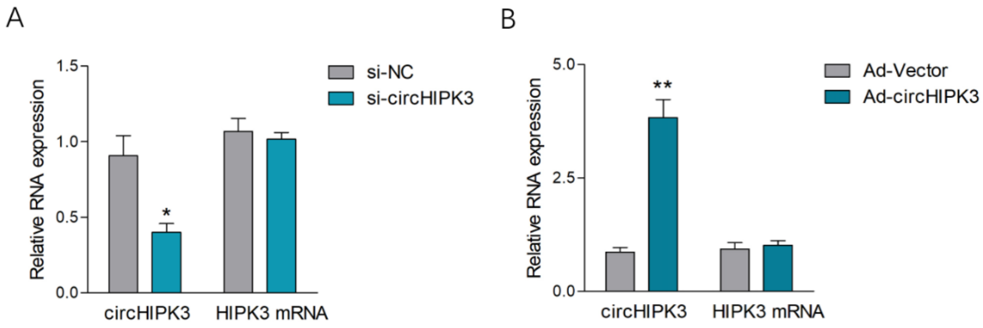

Supplementary Figure 2. Effciency of circHIPK3 overexpression and knockdown.

The knockdown (A) and overexpression (B) of circHIPK3 was determined by qRT-PCR. 60% knockdown and 4-fold overexpression efficiency was achieved, while the expression of linear HIPK3 mRNA was not significantly influenced. (n = 3; *$p < 0.05$, **$p < 0.01$). Data are expressed as the mean ± SEM.

Supplementary Figure 3. Spliced sequence of circHIPK3 (hsa_circ_0000284).

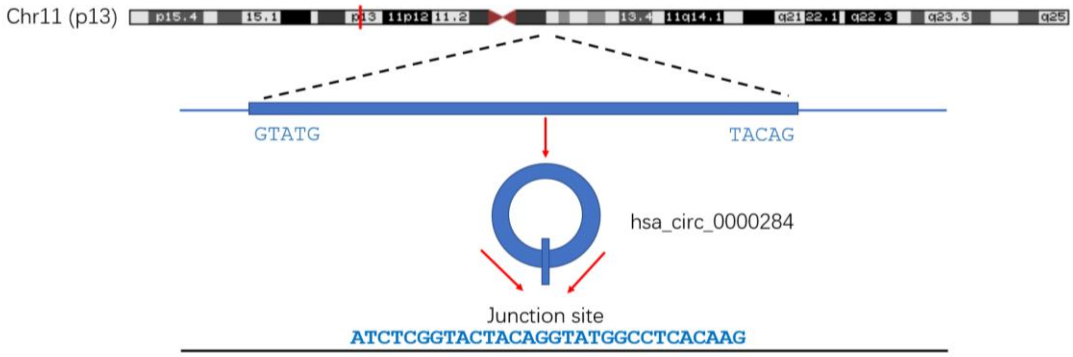

hsa_circ_0000284

GTATGGCCTCACAAGTCTTGGTCTACCCACCATATGTTTATCAAACTCAGTCAAGTGCC
TTTTGTAGTGTGAAGAAACTCAAAGTAGAGCCAAGCAGTTGTGTATTCCAGGAAAGAAA
CTATCCACGGACCTATGTGAATGGTAGAAACTTTGGAAATTCTCATCCTCCCACTAAGG
GTAGTGCTTTTCAGACAAAGATACCATTTAATAGACCTCGAGGACACAACTTTTCATTG
CAGACAAGTGCTGTTGTTTTGAAAAACACTGCAGGTGCTACAAAGGTCATAGCAGCTCA
GGCACAGCAAGCTCACGTGCAGGCACCTCAGATTGGGGCGTGGCGAAACAGATTGCATT
TCCTAGAAGGCCCCCAGCGATGTGGATTGAAGCGCAAGAGTGAGGAGTTGGATAATCAT
AGCAGCGCAATGCAGATTGTCGATGAATTGTCCATACTTCCTGCAATGTTGCAAACCAA
CATGGGAAATCCAGTGACAGTTGTGACAGCTACCACAGGATCAAAACAGAATTGTACCA
CTGGAGAAGGTGACTATCAGTTAGTACAGCATGAAGTCTTATGCTCCATGAAAAATACT
TACGAAGTCCTTGATTTTCTTGGTCGAGGCACGTTTGGCCAGGTAGTTAAATGCTGGAA
AAGAGGGACAAATGAAATTGTAGCAATCAAAATTTTGAAGAATCATCCTTCTTATGCCC
GTCAAGGTCAAATAGAAGTGAGCATATTAGCAAGGCTCAGTACTGAAAATGCTGATGAA
TATAACTTTGTACGAGCTTATGAATGCTTTCAGCACCGTAACCATACTTGTTTAGTCTT
TGAGATGCTGGAACAAAACTTGTATGACTTTCTGAAACAAAATAAATTTAGTCCCCTGC
CACTAAAAGTGATTCGGCCCATTCTTCAACAAGTGGCCACTGCACTGAAAAAATTGAAA
AGTCTTGGTTTAATTCATGCTGATCTCAAGCCAGAGAATATTATGTTGGTGGATCCTGT
TCGGCAGCCTTACAGGGTTAAAGTAATAGACTTTGGGTCGGCCAGTCATGTATCAAAGA
CTGTTTGTTCAACATATCTACAATCTCGGTACTACAG

Supplementary Figure 4

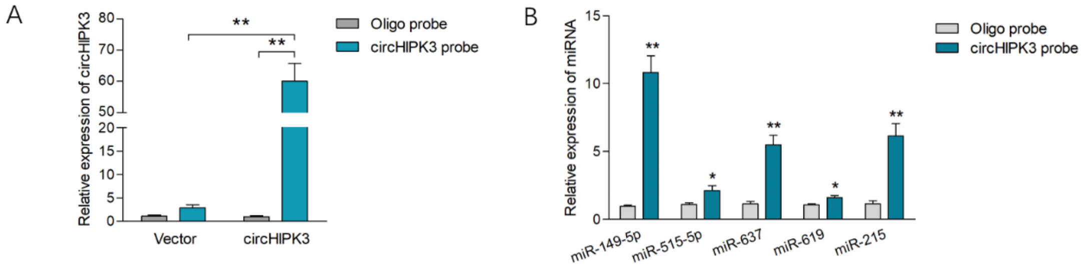

C

Gene	Matching structure	Matching type
circHIPK3<br>miR-149-5p	5' –uucaugcugaucucaAGCCAGag – 3'<br>|||||| 3' –cccucacuucugucggUCGGUCu– 5'	7mer-1a
circHIPK3<br>miR-515-5p	5' –acagaauuguaccacUGGAGAag– 3'<br>|||||| 3' –gucuuucacgaaagaaaACCUCUu– 5'	7mer-1a
circHIPK3<br>miR-637	5' –caagaggauuGGAAG- -CCCAGAGu– 3'<br>||||| ||||||| 3' –gaccuCCUUCccGGGUCUCc– 5'	7mer-m8
circHIPK3<br>miR-619	5' –ucgaGGCACguuugg- -CCAGGUag– 3'<br>||||| |||||| 3' –ugacCCGUGuuuguacaGGUCCAg– 5'	7mer-1a
circHIPK3<br>miR-215	5' –gcauuuccuagaaggCCCCCAGc– 3'<br>||||||| 3' –ugcgucucgggcuuucGGGGGUCa– 5'	7mer-1a

Gene	Matching structure	Matching type
circHIPK3	5' –uucaugcugaucucaAGCCAGag – 3'	7mer-1a
miR-149-5p	3' –cccucacuucugucggUCGGUCu– 5'	
FOXO1	5' –AAGAUUUCCUACAGUAAGCCAGC– 3'	6mer

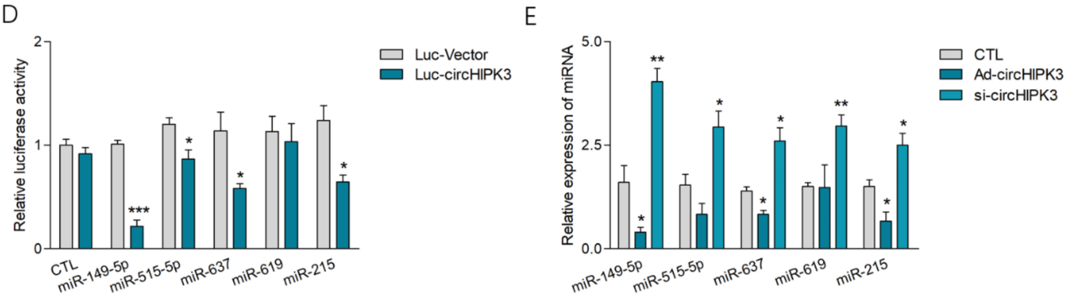

Supplementary Figure 4. MiRNAs selection influenced by circHIPK3.

(A) Pull-down efficiency. Relative levels of circHIPK3 pulled down by the circHIPK3 probe were normalized to the level of circHIPK3 pulled down by an oligo probe. (n=3; **p<0.01). (B) qRT-PCR quantification of the circHIPK3-bound 5 miRNA candidates pulled down in the cell lysates. (n=3; *p<0.05, **p<0.01). (C) At least one miRNA-binding site defined by the Arraystar Proprietary Algorithms was confirmed using the CircInteractome database. The schematic graph illustrated binding sites between circHIPK3 and miR-149-5p, miR-149-5p and FOXO1 mRNA predicted by bioinformatics methods. (D) Relative luciferase activity of circHIPK3 luciferase reporter after co-transfection with indicated 4 miRNA mimics. (n=3; *p<0.05, ***p<0.001). (E) qRT-PCR analysis showed that downregulating circHIPK3 increased miRNAs expression, while upregulating circHIPK3 decreased miRNAs expression, but miR-149-5p was the most significant one. (n=3; *p<0.05, **p<0.01). Data are expressed as the mean ± SEM.

Supplementary Figure 5. Wild type and mutated putative binding sites of circHIPK3 and FOXO1 for luciferase reporter assay.

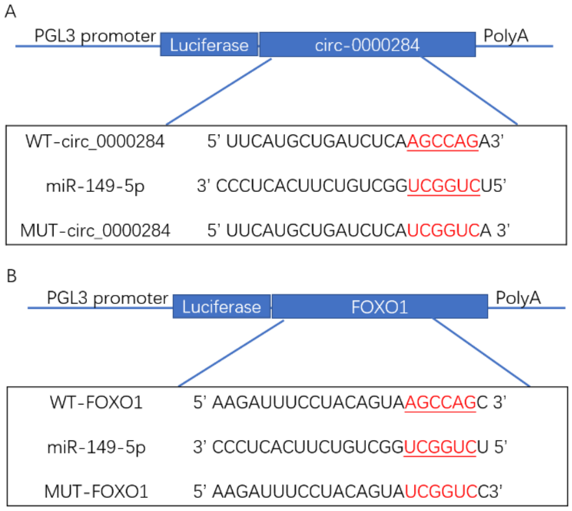

Supplementary Figure 6

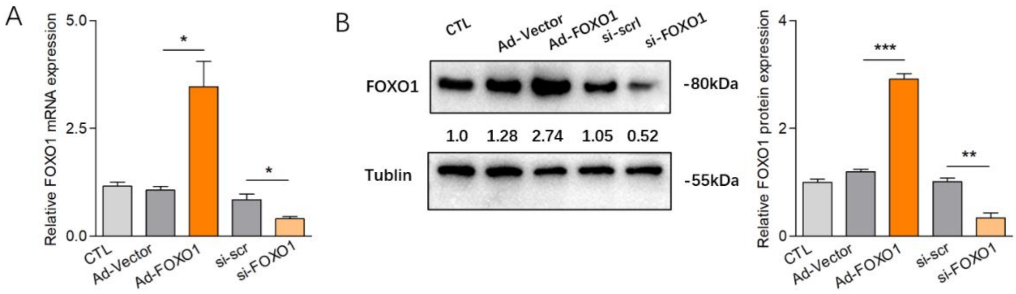

Supplementary Figure 6. Overexpression and silencing of FOXO1.

Both overexpression (A) and silencing (B) of FOXO1 were constructed in RA-FLS, and the stable transfection efficiency of FOXO1 were shown. (n=3; *p<0.05, **p<0.01, ***p<0.001). Data are expressed as the mean ± SEM.

Supplementary Figure 7

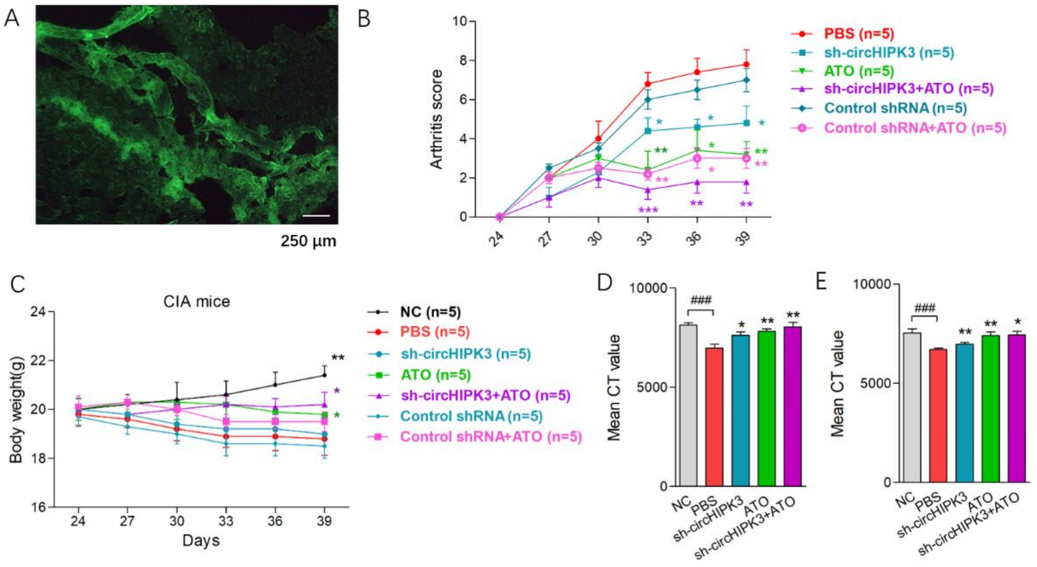

Supplementary Figure 7. Results of shRNA viral vectors control, body-weight of mice, mean values of micro-CT.

(A) AAV-sh-CMV-EGFP viral vectors as a control were intra-articularly administered to CIA mice, and AAV effectively transduced into synovial tissue, displaying a local distribution pattern. (B) AAV vector control AAV-sh-CMV-EGFP management had no significant effect on CIA arthritis and the ATO's curative effect. (n= 5; *p < 0.05, **p < 0.01, ***p < 0.001 versus PBS treatment group). (C) Body-weight loss was recorded to evaluate the curative effects and toxicity of ATO and AAV-sh-circHIPK3 injection. PBS-treated CIA mice showed a signifcant decrease in weight compared with the normal control group. ATO at 2.0 mg/kg/day as well as the combination therapy of ATO and AAV-sh-circHIPK3 injection signifcantly increased body-weight. Furthermore there was no significant difference of body-weight between combination therapy group and normal control group. There was no significant difference of body-weight between shRNA control and PBS-treated group, ATO+ shRNA-control group and ATO group. (n= 5; *p < 0.05, **p < 0.01 versus PBS treatment

group). Mean CT values of paws (D) and knees (E) were evaluated and consistent with the trends toward changes of micro-CT images. (n= 5; *p < 0.05, **p < 0.01 versus PBS treatment group; ### p < 0.001 versus NC). Data are expressed as the mean ± SEM.

Supplementary Figure 8

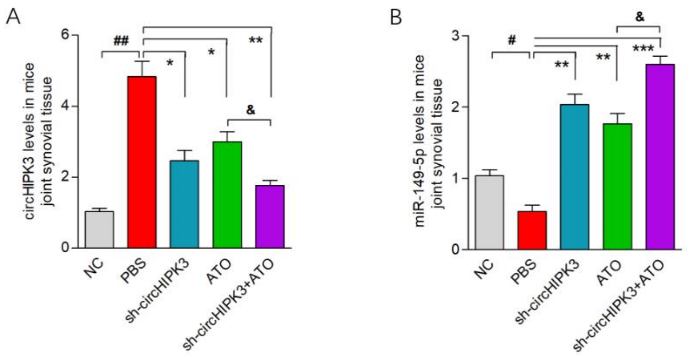

Supplementary Figure 8. Synovial circHIPK3 and miR-149-5p levels in CIA mice joint.
The expression levels of circHIPK3 (A) and miR-149-5p (B) in mice knee joint tissues were detected by qRT-PCR. (n= 5; *p < 0.05, **p < 0.01, ***p < 0.001 versus PBS treatment group; # p < 0.05, ## p < 0.01 versus NC; & p < 0.05 versus ATO group). Data are expressed as the mean ± SEM. NC = normal control group.

Supplementary Table 1 (Table S 1). Primers and probes used in the study.

Gene	Sequence
hsa_circ_0000284	5′-TATGTTGGTGGATCC TGTTCGGCA-3′<br>3′ –TGGTGGGTAGACCAAGACTTGTGA– 5′
HIPK3	5′-ACATTGGAAGAGCATGAGGCAGAGA-3′<br>3′-CTGCTGAAAAGCATCACCACAACCA-5′
miR-149-5p	5′-CCCTCATTCTGTGCCACACTCCAGCTGGG-3′<br>3′ –TGGTGTCGTGGAGTCG-5′
FOXO1	5′ –CAGCAAATCAAGTTATGGAGGA– 3′<br>3′ –TATCATTGTGGGGAGGAGAGTC– 5′
VEGF	5′ –TTCTGGGCTGTTCTCGCTTC– 3′<br>3′ –CTCTCCTCTTCCTTCTCTTCTTCC– 5′
GAPDH	5′ – GCACCGTCAAGGCTGAGAAC – 3′<br>3′ –TGGTGAAGACGCCAGTGGA– 5′
U6	5′-GTAGATACTGCAGTACG-3′<br>3′-ATCGCATGACGTACCTGAGC-5′

circRIP probes	
hsa_circ_0000284	5'ACCAAGACTTGTGAGGCCATACCTGTAGTACCGAGATTGTAGA-3'-biotin
Control	5'CTTCGAGGTACAGGAGTTCGAATGCACCACAAAGACATTCA-3'-biotin
**FISH probes**	
hsa_circ_0000284	5'-CY3-TTGTGAGGCCATACCTGTAGTACCG-3'
**siRNAs**	
si-circ_0000284	5'-TAGAAGACCATGGGGGATA-3'
linearHIPK3	5'-GCUGAUUGAUGCAGAUUUA-3'
si-FOXO1	5'-GCCAAGCCCACAUAAUCATT-3'<br>5'-UGAUUAUGUGGGUCUUGGCTT-3'
si-NC	5'- UUCUCCGAACGUGUCACGU-3'

miR-149-5p mimic	RiboBio
miR-149-5p inhibitor	RiboBio
